# A cross-sectional study on the associations of overweight and smoking with current asthma symptoms in US adults, NHANES 2015–2018

**DOI:** 10.18332/tid/217412

**Published:** 2026-03-11

**Authors:** Wugang Zhou, Jianou Qiao, Hong Zhu

**Affiliations:** 1 Department of Emergency, Shanghai Ninth People's Hospital, School of Medicine, Shanghai Jiao Tong University, Shanghai, China; 2 Department of Respiratory Medicine, Shanghai Ninth People's Hospital, School of Medicine, Shanghai Jiao Tong University, Shanghai, China; 3 Clinical Medical School, Shanghai Ninth People’s Hospital, School of Medicine, Shanghai Jiao Tong University, Shanghai, China

**Keywords:** asthma, overweight, smoking, sex differences, NHANES

## Abstract

**INTRODUCTION:**

Asthma drives significant healthcare use, including frequent emergency department (ED) visits in the US. Modifiable factors like overweight/obesity and smoking exacerbate the disease through inflammation and reduced treatment efficacy. Their combined link to the burden of current symptoms in adults requires further study. This study used nationally representative data to investigate the independent and synergistic associations of overweight and smoking history with current asthma symptoms among United States adults, specifically examining potential gender differences.

**METHODS:**

This cross-sectional study conducted a pooled analysis of secondary data from 1655 participants in the 2015–2016 and 2017–2018 cycles of the National Health and Nutrition Examination Survey (NHANES). The outcome was current asthma symptoms, defined by a positive response to the question ‘Do you still have asthma?’, among those ever diagnosed. Exposures were overweight (body mass index, BMI ≥25 kg/m^2^) and ever smoking (≥100 cigarettes in lifetime). Multivariable logistic regression models adjusted for age and gender were used to assess associations.

**RESULTS:**

Among 1655 participants (mean age 46.5 years), 999 (60.4%) reported current asthma symptoms. In the fully adjusted model, both overweight (adjusted odds ratio, AOR=1.42; 95% CI: 1.12–1.79) and smoking history (AOR=1.27; 95% CI: 1.03–1.57) were independently associated with higher odds of current asthma symptoms. Notable gender differences were observed: these associations were significant and strong in women (overweight, AOR=1.89; smoking, AOR=1.41) but absent in men. No significant interaction between overweight and smoking was detected (interaction p=0.580).

**CONCLUSIONS:**

Overweight and smoking are independent, modifiable predictors associated with current asthma symptoms in US adults, with a disproportionately strong effect observed in women. These findings identify a symptomatic profile that is associated with the likelihood of exacerbations. This profile may help inform clinical attention and future research aimed at reducing asthma morbidity and potential ED visits.

## INTRODUCTION

Asthma-related emergency department (ED) visits represent a critical indicator of disease control failure and impose a substantial burden on healthcare systems worldwide^1^. In the United States alone, approximately 1.6 million asthma-related ED visits and 183000 hospitalizations occurred in 2017^1,2^. These acute care encounters not only reflect suboptimal disease management but also generate substantial healthcare expenditures^2^. Economic analyses indicate that asthma is among the costliest respiratory diseases, with ED services accounting for a significant portion (21.7%) of upper respiratory infection expenditures^3^, highlighting the substantial economic burden of acute respiratory care. Uncontrolled asthma symptoms frequently precipitate acute exacerbations requiring urgent medical attention, with ED visits often serving as sentinel events preceding hospitalizations, missed work or school days^1^, and substantial healthcare utilization that disrupts patients’ quality of life.

Identifying modifiable factors associated with symptomatic exacerbations that may lead to subsequent ED visits is therefore essential for developing effective preventive strategies that address the root causes rather than merely managing acute crises. The relationship between overweight or obesity and asthma involves complex bidirectional pathways. While historically viewed as unidirectional, emerging evidence confirms that elevated body mass index (BMI) directly exacerbates asthma through multiple mechanisms. These include enhanced systemic inflammation with elevated neutrophils and eosinophils, increased airway hyperresponsiveness, exaggerated effects on airway smooth muscle, and potential corticosteroid resistance. Furthermore, obesity-related comorbidities such as metabolic syndrome and visceral adiposity independently impair lung function and asthma-related quality of life, thereby increasing vulnerability to acute exacerbations necessitating emergency care^4,5^. Similarly, tobacco smoke worsens asthma by damaging airways and inducing corticosteroid resistance through mechanisms such as altered glucocorticoid receptors^6,7^. This creates a complex inflammatory environment that drives severe attacks and ED visits, even with optimal treatment.

Current evidence predominantly focuses on direct associations between various factors and asthma diagnoses, symptoms, or healthcare utilization separately^8,9^, creating a conceptual gap in understanding how modifiable factors influence the symptomatic pathway that ultimately drives emergency healthcare seeking, particularly in contemporary, diverse populations. Modifiable factors such as overweight/ obesity and smoking are known to influence asthma pathogenesis and control. The role of current asthma symptom status as a link between these modifiable factors and ED visits is not well understood, particularly how overweight and smoking jointly influence symptom profiles associated with acute care needs, and whether these factors operate independently or synergistically associated with current asthma symptoms, which are a key driver of healthcare utilization. Furthermore, most epidemiological studies have utilized administrative data focusing on formal asthma diagnoses^10,11^, potentially missing the substantial proportion of individuals experiencing significant asthma symptoms who have not received formal diagnosis, but nonetheless experience impaired quality of life and may utilize emergency services for symptom relief.

This study therefore aimed to investigate the independent and joint associations of overweight and smoking history with current asthma symptoms among US adults, utilizing nationally representative data. A particular focus was placed on examining potential differences in these associations by gender. By focusing on current symptom status – a direct precursor to emergency care utilization – this study addresses a gap in understanding how these modifiable factors relate to the symptomatic burden that often drives healthcare seeking.

## METHODS

### Study design and data source

This cross-sectional study involved a pooled analysis of secondary data from the National Health and Nutrition Examination Survey (NHANES) for the cycles 2015–2016 and 2017–2018. NHANES employs a complex, multistage, probability sampling design to obtain a nationally representative sample of the non-institutionalized civilian population in the United States. Data are collected through structured household interviews and standardized physical examinations at mobile examination centers. For this analysis, data on asthma diagnosis and symptoms (questionnaire MCQ series) and smoking history (questionnaire SMQ series) were based on self-report. Height and weight were measured by trained health technicians to calculate body mass index (BMI, kg/m^2^).

### Eligibility criteria

This study included non-institutionalized civilian adults (aged ≥20 years) who participated in the NHANES 2015–2016 or 2017–2018 cycles and had complete data on the variables of interest: current asthma symptoms, body mass index (BMI), smoking status, and sampling weights. Participants with missing data on any of these key variables were excluded.

### Study population

**Figure 1:**
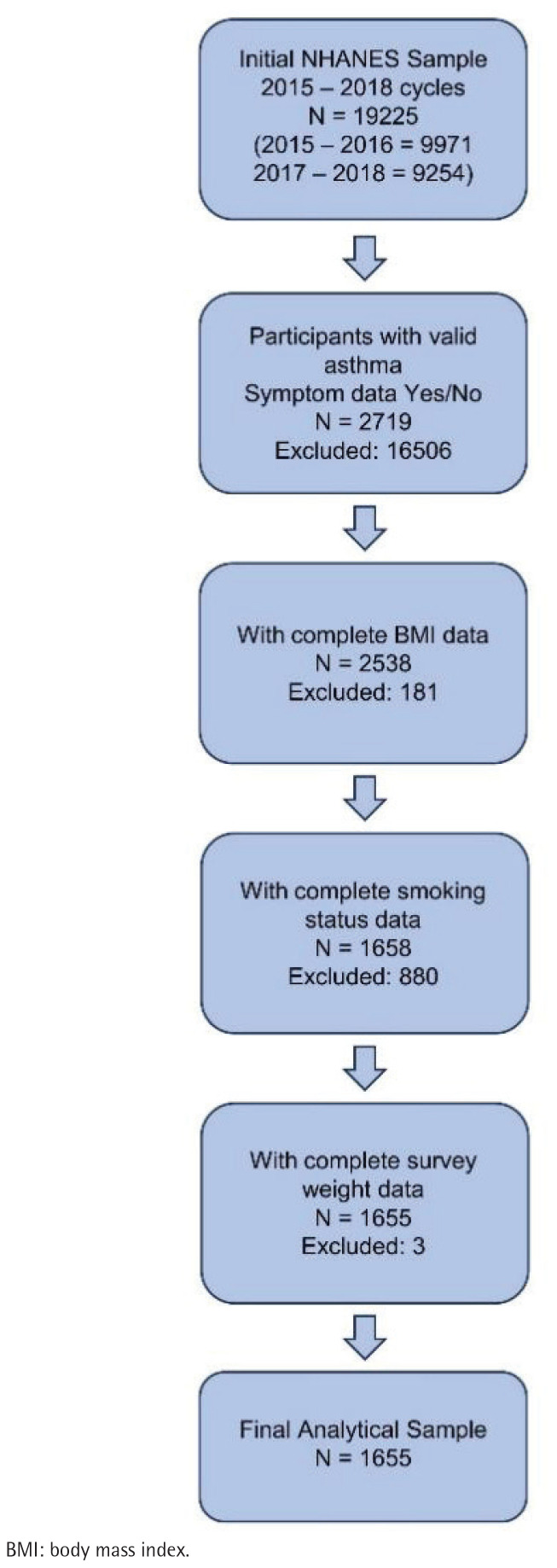
Flowchart of participant selection from the NHANES 2015–2018 cycles

From the pooled data, participants meeting the eligibility criteria were included. The initial analytical sample comprised adult participants aged ≥20 years from the pooled NHANES 2015–2016 and 2017–2018 data. Due to the secondary analysis nature of this study, intermediate counts for each exclusion step were not archived; however, the specific criteria and sequence are detailed below. This research sequentially excluded individuals with missing data on the following key variables: 1) current asthma symptoms (MCQ035); 2) body mass index (BMI) for determining overweight status; 3) smoking status (SMQ020); and 4) interview examination weights (WTINT2YR). These exclusions resulted in a final analytic sample of 1655 participants for all subsequent analyses. A detailed flowchart illustrating participant selection is provided in Figure 1.

### Outcome variable

The primary outcome was current asthma symptoms, derived from a two-step assessment. First, participants were identified as having ever received an asthma diagnosis based on MCQ010: ‘Has a doctor or other health professional ever told you that you have asthma?’. Those responding affirmatively were then asked (MCQ035): ‘Do you still have asthma?’. Participants responding ‘Yes’ were classified as having current asthma symptoms, while those responding ‘No’ were classified as having a historical diagnosis without current symptoms.

### Primary exposure variables

*Overweight status*

Participants with a BMI ≥25.0 kg/m^2^ were classified as being overweight or obese. Those with BMI <25.0 kg/m^2^ constituted the normal-weight reference group.

*Smoking status*

Based on SMQ020, participants who reported having smoked at least 100 cigarettes in their lifetime were classified as ‘ever smokers’; all others were classified as ‘never smokers’.

### Covariates

Covariate selection was guided by established clinical knowledge and prior research on asthma determinants^8,9^. The primary analysis adjusted for two key demographic confounders: 1) age (modeled as a continuous variable in years), given the known variations in asthma prevalence and presentation across adulthood; and 2) gender (categorized as male/ female), due to well-documented sex differences in asthma epidemiology and response to environmental exposures. These covariates were included in adjusted models (Models 3 and 4) to isolate the independent effects of overweight status and smoking status on current asthma symptoms.

### Statistical analysis

All statistical analyses were conducted using R software version 4.5.1^12^ (R Foundation for Statistical Computing, Vienna, Austria). The following R packages were utilized for specific analytical tasks: *tidyverse* (version 2.0.0) for data manipulation, management, and visualization;^13^ survey (version 4.4.8) for handling complex survey design elements;^14^ and *gtsummary* (version 2.4.0) for generating publication-ready tables of descriptive statistics and regression results^15^.

This research employed sampling weights (WTINT2YR) to account for the complex survey design and enhance population representativeness in all analyses. Descriptive statistics were computed as means with standard deviations (SD) for continuous variables and as frequencies with percentages for categorical variables, stratified by current asthma symptom status. Group comparisons were performed using independent samples t-tests for continuous variables and chi-squared tests for categorical variables.

The primary analytical approach involved multivariable logistic regression to examine the independent and joint associations of overweight status and smoking history with current asthma symptoms. Four sequential models were constructed: Model 1 assessed the unadjusted association for overweight status; Model 2 assessed the unadjusted association for smoking status; Model 3 included both overweight and smoking status with adjustment; and Model 4 further adjusted for age (modeled as a continuous variable) and gender. Results are presented as odds ratios (ORs) with their corresponding 95% confidence intervals (CIs). Statistical significance was defined as a two-sided p<0.05.

Regarding data handling, participants with missing data on any of the key variables (current asthma symptoms, overweight status, smoking status, or sampling weights) were excluded from the analysis, as detailed in the Study population section. While the survey package in R was available, the primary dataset lacked complete information on the complex survey design variables (strata and clusters). Therefore, to maintain a consistent and robust analytical approach across all models, the primary analyses utilized weighted logistic regression with interview weights, a well-established method for deriving nationally representative estimates from NHANES data.

### Ethical considerations

The survey protocol for NHANES was approved by the National Center for Health Statistics Research Ethics Review Board, and all participants provided written informed consent^16^. Because this analysis used publicly available, de-identified data, it was considered exempt from further institutional review board approval and was classified as non-human subjects’ research.

## RESULTS

### Baseline characteristics of the study population

The final analytical sample comprised 1655 participants from the NHANES 2015–2018 cycles. Baseline characteristics stratified by the presence of current asthma symptoms are summarized in Table 1. A total of 999 participants (60.4%) reported current asthma symptoms. Participants with current asthma symptoms were significantly more likely to be female (64.9% vs 46.2%, p<0.001), older (mean age 48.5 ± 18.5 vs 43.6 ± 18.6 years, p<0.001), and have a higher body mass index (BMI: 32.0 ± 9.0 vs 29.6 ± 8.1 kg/m^2^, p<0.001; see unadjusted association in Table 2) compared to those without symptoms. The prevalence of overweight was significantly higher in the symptomatic group (78.0% vs 68.9%, p<0.001), while a non-significant trend was observed for a higher prevalence of ever smoking (47.6% vs 43.0%, p=0.069; Table 2).

**Table 1:** Baseline characteristics of the study population by current asthma symptom status, NHANES 2015–2018 (N=1655)

*Variables*	*Symptomatic (N=999)* *n (%)*	*Asymptomatic (N=656)* *n (%)*	*p*
Overweight	779 (78.0)	452 (68.9)	<0.001
Ever smoke	476 (47.6)	282 (43.0)	0.0694
Female	648 (64.9)	303 (46.2)	<0.001
Male	351 (35.1)	353 (53.8)	<0.001
Age (years), mean ± SD	48.5 ± 18.5	43.6 ± 18.6	<0.001
BMI (kg/m^2^), mean ± SD	32.0 ± 9.0	29.6 ± 8.1	<0.001
**Proportion with asthma symptoms by gender**			
Gender	Total	With symptoms	Percentage
Female	951	648	68.1
Male	704	351	49.9
Total	1655	999	60.4

**Table 2:** Logistic regression analysis for the association of overweight and smoking with current asthma symptoms, NHANES 2015–2018 (N=1655)

*Univariate*	*Variable*	*OR (95% CI)*	*p*
Overweight Model 1	Overweight	1.60 (1.28–2.00)	<0.001
Smoking Model 2	Ever smoker	1.21 (0.99–1.47)	0.0628
***Multivariable***	***Variable***	***AOR (95% CI)***	***p***
Model 3	Overweight	1.59 (1.27–1.98)	<0.001
	Ever smoker	1.19 (0.97–1.45)	0.0920
Model 4	Overweight	1.42 (1.12–1.79)	0.0033
	Ever smoker	1.27 (1.03–1.57)	0.0283
	Age (years)	1.01 (1.01–1.02)	<0.001
	Male	0.45 (0.37–0.56)	<0.001

AOR: adjusted odds ratio. Model 1 is the unadjusted model for overweight. Model 2 is the unadjusted model for smoking. Model 3 is adjusted for overweight and smoking. Model 4 is further adjusted for age and gender.

### Associations of overweight, smoking, and current asthma symptoms

The results of the logistic regression analyses are presented in Table 2. In univariate analyses, overweight status was significantly associated with current asthma symptoms (OR=1.60; 95% CI: 1.28–2.00, p<0.001), while the association for smoking history was not statistically significant (OR=1.21; 95% CI: 0.99–1.47, p=0.063). In the multivariable model adjusted for both overweight and smoking (Model 3), the association for overweight remained significant (OR=1.59; 95% CI: 1.27–1.98, p<0.001). After further adjustment for age and gender (Model 4), both overweight (AOR=1.42; 95% CI: 1.12–1.79, p=0.003) and smoking history (AOR=1.27; 95% CI: 1.03–1.57, p=0.028) demonstrated statistically significant, independent associations with higher odds of current asthma symptoms. Older age (AOR=1.01; 95% CI: 1.01–1.02, p<0.001) and male gender (AOR=0.45; 95% CI: 0.37–0.56, p<0.001) were also significantly associated with the outcome.

### Subgroup analysis by gender

Stratified analyses by gender revealed notable differences (Table 3). Among females, both overweight (AOR=1.89; 95% CI: 1.38–2.59, p<0.001) and smoking history (AOR: 1.41; 95% CI: 1.05–1.88, p=0.021) were significantly associated with current asthma symptoms. Conversely, among males, neither overweight (AOR=1.00; 95% CI: 0.71–1.41, p=0.990) nor smoking history (AOR=1.03; 95% CI: 0.74–1.42, p=0.869) showed a significant association; however, increasing age was a strong predictor (AOR=1.02; 95% CI: 1.01–1.03, p<0.001). The formal test for interaction between overweight and smoking status was not statistically significant (interaction p =0.580), indicating no evidence of a synergistic effect on the multiplicative scale (Table 4).

**Table 3:** Subgroup analysis by gender: adjusted associations of overweight and smoking with current asthma symptoms, NHANES 2015–2018

*Variable*	*Female subgroup*		*Male subgroup*	
	*AOR (95% CI)*	*p*	*AOR (95% CI)*	*p*
Overweight	1.89 (1.38–2.59)	<0.001	1.00 (0.71–1.41)	0.990
Ever smoker	1.41 (1.05–1.88)	0.021	1.03 (0.75–1.41)	0.869
Age (years)	1.00 (0.99–1.01)	0.590	1.02 (1.01–1.03)	<0.001
**Model fit**				
Null deviance	1190.3		975.95	
Residual deviance	1166.9		945.02	
AIC	1174.9		953.02	

AOR: adjusted odds ratio. A lower Akaike information criterion (AIC) value suggests a better-fitting model.

**Table 4:** Analysis of interaction between overweight and smoking status on current asthma symptoms, NHANES 2015–2018 (N=1655)

*Variable*	*Estimate*	*SE*	*z*	*Pr(>|z|)*	*AOR (95% CI)*
Intercept	-0.127	0.175	-0.730	0.466	-
Overweight (yes vs no)	0.405	0.157	2.585	0.010	1.50 (1.10–2.04)
Smoking status (ever vs never)	0.333	0.205	1.625	0.104	1.40 (0.93–2.09)
Age (years)	0.011	0.003	3.712	<0.001	1.01 (1.01–1.02)
Gender (Male)	-0.788	0.106	-7.439	<0.001	0.45 (0.37–0.56)
Interaction: Overweight × Smoking	-0.131	0.236	-0.553	0.580	0.88 (0.55–1.39)
**Model fit and Conclusion** **AIC:** 2140.4 **Interaction p-value:** 0.580 **Conclusion:** No significant interaction effect.					

AOR: adjusted odds ratio. SE: standard error. AIC: Akaike information criterion.

Sensitivity analysis (exposure definition) A sensitivity analysis defining exposure as obesity (BMI ≥30 kg/m^2^) instead of overweight yielded consistent findings. Obesity remained significantly associated with current asthma symptoms (AOR=1.38; 95% CI: 1.13–1.70, p=0.002) in the fully adjusted model, reinforcing the robustness of the primary results (Table 5).

**Table 5:** Sensitivity analysis: association of obesity (as exposure) and smoking with current asthma symptoms, NHANES 2015–2018 (N=1655)

*Variable*	*Estimate*	*SE*	*z*	*Pr(>|z|)*	*AOR (95% CI)*
Intercept	-0.030	0.152	-0.197	0.844	-
Overweight (yes vs no)	0.325	0.104	3.115	0.002	1.38 (1.13–1.70)
Smoking status (ever vs never)	0.218	0.108	2.018	0.044	1.24 (1.01–1.54)
Age (years)	0.012	0.003	4.083	<0.001	1.01 (1.01–1.02)
Gender (male)	-0.764	0.106	-7.189	<0.001	0.47 (0.38–0.57)

AOR: adjusted odds ratio. SE: standard error. AIC: Akaike information criterion.

## DISCUSSION

This nationally representative study identifies overweight and smoking history as independent, modifiable factors associated with for current asthma symptoms – a potent precursor to asthma-related ED visits. Our analysis reveals that these factors are associated with a greater burden of active symptomatology, which in turn is the primary driver of acute exacerbations necessitating emergency care. A profound gender disparity was uncovered, with the associations of both overweight and smoking being strongly operative in women but absent in men, suggesting that this symptom-associated profile is gender-specific. The absence of a synergistic interaction indicates that the contributions of overweight and smoking to this symptomatic pathway are additive. Collectively, these findings pinpoint overweight and smoking, particularly in women, as key leverage points for efforts aimed at preventing the symptomatic escalation that culminates in ED utilization.

The identified independent association between overweight and current asthma symptoms corroborates and extends a substantial body of epidemiological evidence^4,5^. The observed significant increase in the odds of current asthma symptoms associated with overweight aligns with the prevailing pathophysiological paradigm, which posits that adipose tissue, particularly visceral fat, is not an inert storage depot but a prolific endocrine organ^17^. Its secretion of pro-inflammatory adipokines (e.g. leptin, TNF-α) creates a state of systemic inflammation that can exacerbate underlying airway inflammation and bronchial hyper-responsiveness^4,18^. Concurrently, mechanical effects of excess weight, including reduced lung volumes and increased work of breathing, likely contribute to symptom perception and burden, independent of inflammatory pathways^5^. The persistence of this association after accounting for smoking and demographic factors underscores its robustness.

An important contribution of this analysis is the demonstration of smoking history as an independent predictor of current asthma symptoms upon adjustment. While the detrimental role of smoking in asthma control and exacerbation risk is incontrovertible^6^, its effect on sustaining the symptomatic state in a general population sample underscores a significant, and often addressable, public health burden. The mechanisms are multifactorial, encompassing direct epithelial injury, impaired ciliary clearance, a shift towards treatment-resistant neutrophilic airway inflammation, and the induction of glucocorticoid receptor dysfunction^19^, collectively fostering a milieu of persistent symptoms and diminished therapeutic response^7^.

The most compelling and nuanced finding of this study is the profound gender effect modification. The fact that the associations of both overweight and smoking with symptoms were significant and substantial in women but entirely absent in men suggests fundamentally distinct pathophysiological landscapes. This disparity may be mediated by sexual dimorphism in immune responses^20^, the influence of sex hormones (e.g. estrogen’s potential to potentiate T-helper-2 responses)^21^, or gender-specific differences in body fat distribution and its associated inflammatory profile^22,23^. In stark contrast, the emergence of advancing age as a strong associated factor exclusively in men warrants investigation into male-specific, age-related declines in lung function or other senescent pathways.

The absence of a statistically significant interaction between overweight and smoking suggests that their combined impact on asthma symptoms is best described by an additive model rather than a synergistic one on the multiplicative scale. This implies that the deleterious effects of smoking and overweight operate through largely independent biological pathways to influence symptom status. From a public health perspective, this is an encouraging finding, as it suggests that addressing either of these factors could be beneficial, regardless of an individual’s status regarding the other.

### Strengths and limitations

The interpretations of our findings must be contextualized within the study’s methodological framework. Principal strengths include the utilization of a large, nationally representative sample, which enhances the generalizability of our conclusions to the non-institutionalized adult population. The operational definition of ‘current asthma symptoms’ effectively identifies a high-risk population burdened by active disease, which constitutes the proximate determinant and clinical precursor to acute exacerbations that precipitate ED visits. Furthermore, a rigorous analytical approach was employed, encompassing multivariable adjustment, formal testing for interaction, and sensitivity analyses, which collectively bolster the validity of the inferences drawn.

Notwithstanding these strengths, several limitations merit consideration. The cross-sectional design inherently prohibits causal inference. The issue of reverse causality cannot be dismissed; for instance, poorly controlled asthma may lead to physical activity limitation and subsequent weight gain. The use of measured BMI strengthens the assessment of weight status; however, the reliance on self-reported data for smoking and asthma diagnosis introduces potential for recall or misclassification bias. This study adjusted for key demographic confounders, yet the possibility of residual confounding from unmeasured variables, such as detailed socioeconomic status, occupational exposures, or medication adherence, cannot be ruled out. Furthermore, due to the archival nature of the secondary data analysis, the precise number of participants excluded at each step of the sample selection process (e.g. for missing data on specific variables) was not retained. While the complete exclusion criteria are transparently reported, the inability to provide these exact counts limits a granular assessment of potential selection bias at each filtration stage. Finally, the interpretation of BMI is limited by its inability to distinguish between muscle and fat mass or to reflect body fat distribution, a consideration that is especially pertinent given the observed gender differences.

### Implications

These findings carry salient implications for both clinical practice and public health strategy. The collective evidence from this study highlights the need to investigate whether integrating proactive weight management support and smoking cessation programs into comprehensive asthma care, particularly for women who exhibited the strongest associations, can improve asthma outcomes. From a public health perspective, population-level campaigns aimed at reducing the prevalence of overweight and smoking might help mitigate the burden of symptomatic asthma. Future longitudinal and intervention studies are needed to determine whether addressing these factors can improve asthma outcomes. From a public health research perspective, investigating whether population-level changes in the prevalence of overweight and smoking are associated with variations in the burden of symptomatic asthma represents a valuable direction for future study.

## CONCLUSIONS

This study provides robust, nationally representative evidence that overweight and smoking history are significant, independent associated with current asthma symptoms in adults. The identification of a distinct profile characterized by female gender, overweight, and smoking history, which is strongly associated with current asthma symptoms, points to a subgroup that may benefit from heightened clinical awareness and further evaluation. By delineating the association between these modifiable factors and active symptomatology – a key driver of healthcare utilization – this analysis highlights the potential importance of considering weight management and smoking status in the context of asthma symptom burden. These findings underscore the need for future research to evaluate whether interventions targeting these factors can effectively mitigate asthma symptoms and reduce the need for acute care. Ultimately, these findings provide a basis for future research to explore whether addressing these modifiable factors can mitigate the symptomatic burden of asthma and reduce reliance on emergency healthcare services.

## Data Availability

Data sharing is not applicable to this article as no new data were created.
